# Dissecting specific Wnt components governing osteogenic differentiation potential by human periodontal ligament stem cells through interleukin-6

**DOI:** 10.1038/s41598-023-35569-8

**Published:** 2023-06-03

**Authors:** Medania Purwaningrum, Cecilia M. Giachelli, Thanaphum Osathanon, Sirirat Rattanapuchpong, Chenphop Sawangmake

**Affiliations:** 1 The International Graduate Program of Veterinary Science and Technology (VST), Faculty of Veterinary Science, Chulalongkorn University, Bangkok, 10330 Thailand; 2 Veterinary Stem Cell and Bioengineering Innovation Center (VSCBIC), Faculty of Veterinary Science, Chulalongkorn University, Bangkok, 10330 Thailand; 3 Department of Biochemistry, Faculty of Veterinary Medicine, Universitas Gadjah Mada, Yogyakarta, 55281 Indonesia; 4 Veterinary Stem Cell and Bioengineering Research Unit, Faculty of Veterinary Science, Chulalongkorn University, Bangkok, 10330 Thailand; 5 Department of Bioengineering, University of Washington, Seattle, WA 98195 USA; 6 Department of Anatomy, Faculty of Dentistry, Chulalongkorn University, Bangkok, 10330 Thailand; 7 Dental Stem Cell Biology Research Unit, Faculty of Dentistry, Chulalongkorn University, Bangkok, 10330 Thailand; 8 Center of Excellence in Regenerative Dentistry (CERD), Chulalongkorn University, Bangkok, 10330 Thailand; 9 Academic Affairs, Faculty of Veterinary Science, Chulalongkorn University, Bangkok, 10330 Thailand; 10 Department of Pharmacology, Faculty of Veterinary Science, Chulalongkorn University, Bangkok, 10330 Thailand

**Keywords:** Cell biology, Molecular biology, Stem cells

## Abstract

Periodontal ligament stem cells (PDLSCs) play a significant role on periodontal tissue and alveolar bone homeostasis. During inflammation, interleukin (IL)-6 serves as one of key cytokine players controlling tissue reaction as well as alveolar bone tissue remodeling. It is believed that periodontal tissue inflammation causes periodontium degradation, especially alveolar bone. However, in this study, we show that an inflammatory mediator, IL-6, may serve another direction on alveolar bone homeostasis during inflammatory condition. We found that, IL-6 at 10 and 20 ng/mL was not cytotoxic and dose-dependently exerted beneficial effects on osteogenic differentiation of human PDLSCs (hPDLSCs), as demonstrated by increased alkaline phosphatase activity, mRNA expression of osteogenic markers, and matrix mineralization. The presence of physiological and inflammatory level of IL-6, the osteogenic differentiation potential by hPDLSCs was enhanced by several possible mechanisms including transforming growth factor (TGF), Wnt, and Notch pathways. After in-depth and thorough exploration, we found that Wnt pathway serves as key regulator controlling osteogenic differentiation by hPDLSCs amid the IL-6 presentation. Surprisingly, apart from other mesenchymal stem cells, distinct Wnt components are employed by hPDLSCs, and both canonical and non-canonical Wnt pathways are triggered by different mechanisms. Further validation by gene silencing, treatment with recombinant Wnt ligands, and β-catenin stabilization/translocation confirmed that IL-6 governed the canonical Wnt/β-catenin pathway via either WNT2B or WNT10B and employed WNT5A to activate the non-canonical Wnt pathway. These findings fulfill the homeostasis pathway governing periodontal tissue and alveolar bone regeneration and may serve for further therapeutic regimen design for restoring the tissues.

## Introduction

Oral and periodontal diseases can result in destruction of the alveolar bone and periodontal tissue, especially in severe and chronic cases^1–5^. Based on the severity, periodontal disease can be classified from type I to V, that is from gingivitis, mild to severe periodontitis^6^. Based on cause, periodontitis can be classified into inflammatory that caused by bacterial infection, whereas *Porphyromonas gingivalis* is one of the major pathogens, and non-inflammatory periodontitis^7^. It is challenging to repair the inflammation in periodontal tissues, that causes periodontal tissues destruction and bone resorption extensively^8–10^. In this context, homeostasis refers to the balance of regeneration and remodeling of oral and periodontal tissues versus the extent of disease^11–13^, which is largely influenced by the state of inflammation^14–20^. Inflammation is regulated by both pro- and anti-inflammatory cytokines^21–23^. Interleukin (IL)-6 is a pro-inflammatory cytokine that plays a pivotal role in inflammation and cellular properties, such as the multilineage differentiation potential of mesenchymal stem cells (MSCs)^20,23–27^. Human periodontal ligament stem cells (hPDLSCs), which maintain the alveolar bone and periodontal tissues, exhibit multilineage differentiation potential toward endodermal, mesodermal, and ectodermal lineages, suggesting cell plasticity^28–32^. hPDLSCs were firstly isolated in 2004^33^, and expressed profile that similar to MSCs including CD13, CD29, CD44, CD73, CD90, CD105, CD106, CD146, CD166, STRO-1, STRO-3, SSEA-1, SSEA-4, *OCT4*, *REX1*, *NANOG*, *Ki67* and lacked of CD11b, CD14, CD31, CD33, CD34, CD45, CD133, CD144, CD79, CD19, and HLA-DR^32,34–36^. Upon exposure to various stimuli, hPDLSCs are crucial for maintenance and homeostasis of surrounding tissues^37,38^. IL-6, which is produced and secreted in response to mechanical and inflammatory stimuli, governs the remodeling of oral and periodontal tissues^24,25,39^, and the stemness and osteogenic differentiation. This suggests the potential application of IL-6 in therapeutic regimens^20,40–42^. Although the effect of IL-6 on the osteogenic differentiation potential of hBM-MSCs is well established, relatively few studies have investigated the effects of IL-6 on hPDLSCs in the alveolar bone and periodontium, especially during disease progression and inflammation^11^. In addition, hPDLSCs regulate regeneration and remodeling of the alveolar bone and periodontium^39,43^, suggesting the importance of the interplay between hPDLSCs and IL-6 in tissue homeostasis.

In terms of tissue regeneration, the homeostasis pathways of periodontal tissue and alveolar bone serve as key components on natural periodontium tissue regeneration and tissue engineering designated for reconstructing the damaged tissue. It is interesting that amid the inflammatory condition of particular tissue, the group of inflammatory or pro-inflammatory cytokines may trigger the distinct mechanisms which help maintain the tissue restoration rather than destruction. The Wnt signaling pathway has been known as an essential pathway in the osteogenic differentiation for promoting bone formation by MSCs^44,45^. In addition, Wnt pathway is crucial in the process of homeostasis and pathological process of periodontal tissue and alveolar bone regeneration^9,10,46,47^. Here, in this study, the relationship between MSCs supporting periodontal tissue and alveolar bone restoration, hPDLSCs, and key inflammatory cytokine, IL-6, were thoroughly explored to understand the outcome of the phenomenon and the potential signaling pathways underlying the findings. This discovery will fulfill the picture of homeostasis pathway used for naturally governing periodontal tissue and alveolar bone regeneration and will serve as a key fundamental knowledge for designing the therapeutic regimens for periodontium regeneration.

## Results

### Isolated hPDLSCs exhibit MSC-like properties

**Figure 1:**
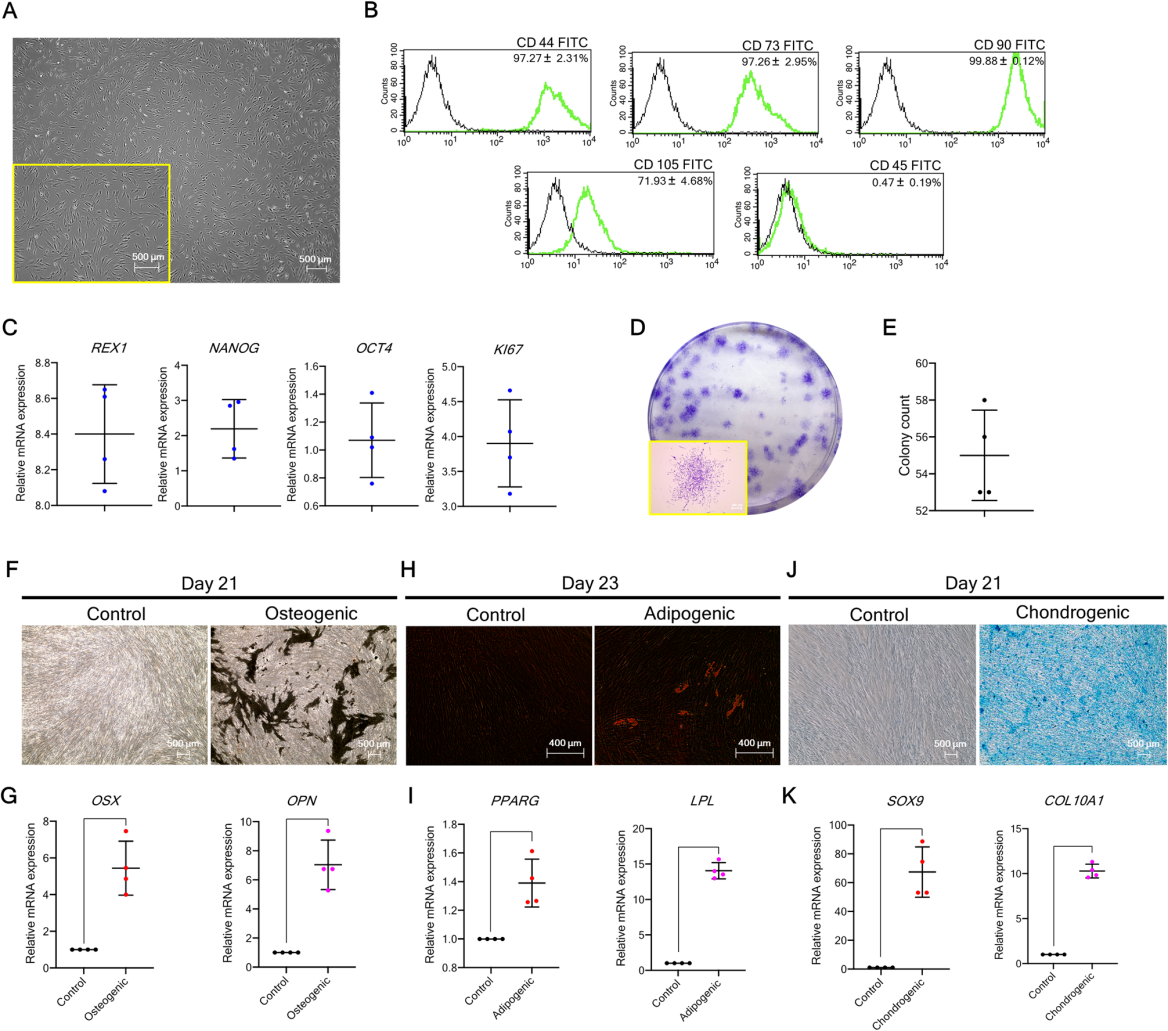
hPDLSC characterization. (**A**) Morphological appearance of the isolated hPDLSCs. (**B**) Flow cytometry was performed to assess the mesenchymal stem cell-related surface markers (CD44, CD73, CD90, and CD105) and hematopoietic cell surface marker (CD45). (**C**) RT-qPCR was performed to quantify mRNA expression of markers relating to stemness (*REX1, NANOG,* and *OCT4*) and proliferatiion *(KI67*). (**D**,**E**) Colony appearance and total colony count are presented. (**F**,**H**,**J**) Multilineage differentiation potential toward the osteogenic, adipogenic, and chondrogenic lineages is demonstrated by staining with *Von Kossa*, Oil Red O, and Alcian Blue, respectively. (**G**,**I**,**K**) mRNA expression of markers of the osteogenic, adipogenic, and chondrogenic lineages, respectively (n = 4). Bars indicate significant differences (*p* < 0.05).

In two-dimensional (2D) culture, hPDLSCs exhibited a fibroblast-like morphology and tended to adhere to the culture dish (Fig. 1A). Flow cytometry confirmed expression of the MSC-related surface markers CD44, CD73, CD90, and CD105, but not the hematopoietic cell surface marker CD45 (Fig. 1B), in addition to the stemness-related markers *REX1, NANOG,* and *OCT4* as well as the proliferative marker *KI67* (Fig. 1C). The colony-forming capability of hPDLSCs was also observed (Fig. 1D,E). Staining and mRNA marker expression demonstrated the multilineage differentiation potential of hPDLSCs toward osteogenic, adipogenic, and chondrogenic lineages (Fig. 1F–K) (n = 4). Collectively, these results highlight the characteristics shared by MSCs and hPDLSCs.

### Physiological and inflammatory levels of IL-6 promote osteogenic differentiation potential of hPDLSCs in vitro

**Figure 2:**
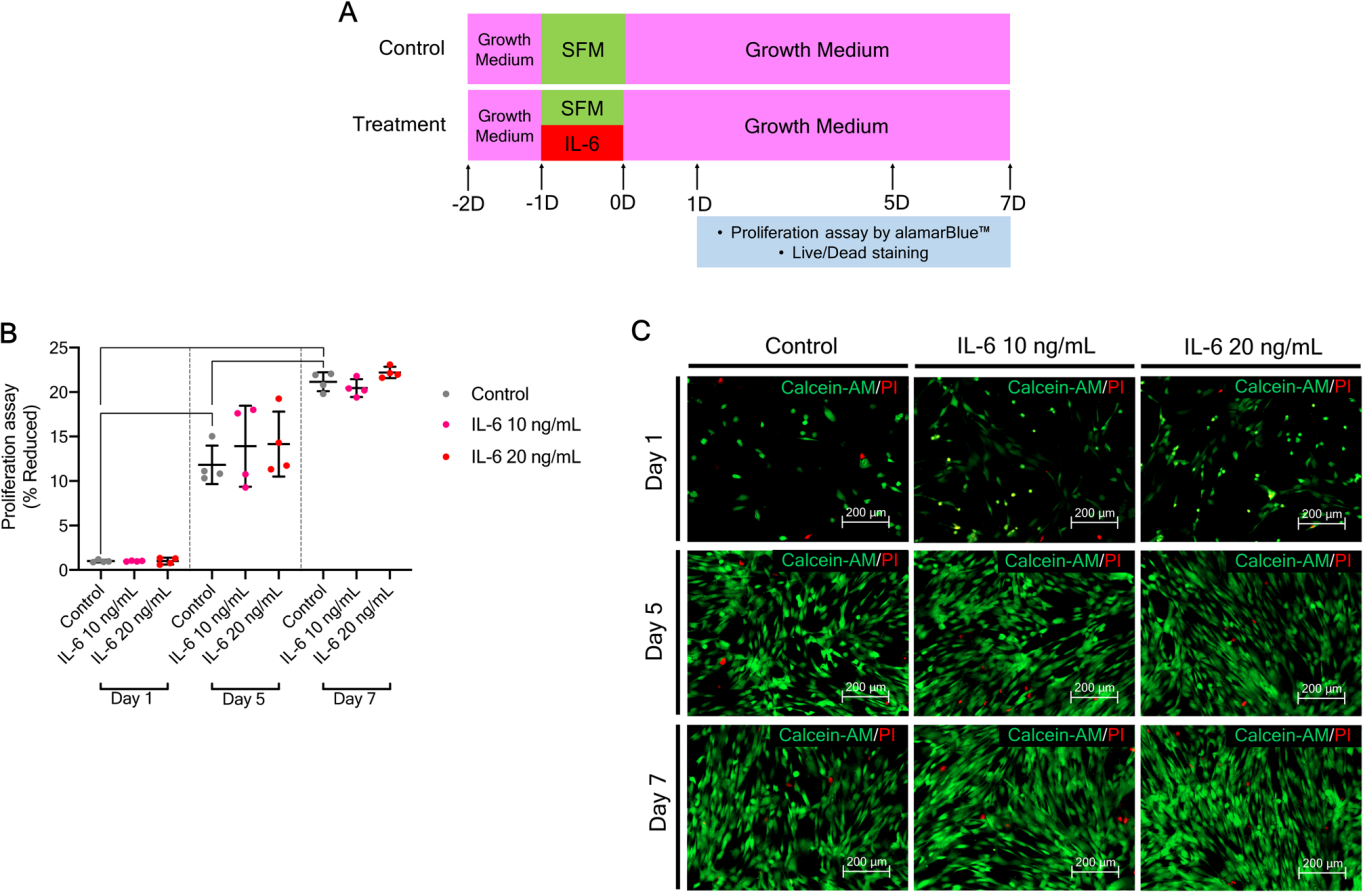
Proliferation and viability of hPDLSCs upon IL-6 treatment. (**A**) Schematic presentation of the experiment. (**B**) Proliferation of hPDLSCs upon IL-6 treatment (10 and 20 ng/mL) at days 1, 5, and 7, as determined by alamarBlue™ staining. (**C**) Viability of hPDLSCs upon IL-6 treatment (10 and 20 ng/mL) at days 1, 5, and 7 (calcien AM/propidium iodide) (n = 4). Bars indicate significant differences (p < 0.05).

The effects of IL-6 at 10 and 20 ng/mL on the osteogenic differentiation potential of hPDLSCs in vitro were assessed. The results of the cell proliferation and viability assays showed that IL-6 was not cytotoxic (Fig. 2A). hPDLSCs were maintained in culture medium pre-treated with and without IL-6 at 10 and 20 ng/mL. Proliferation and viability were determined with the alamarBlue assay and staining of viable and dead cells on days 1, 5, and 7. The results of the alamarBlue assay showed no significant difference in cell proliferation on days 1, 5, and 7 between the control group and the groups treated with IL-6 at 10 and 20 ng/mL (Fig. 2B), while the cell viability assay reveled no difference in the populations of viable and dead cells at every time point (Fig. 2C) (n = 4). Since IL-6 at 10 and 20 ng/mL was not cytotoxic, these dosages were used to assess the effects of IL-6 on the osteogenic differentiation potential of hPDLSCs in vitro*.*

**Figure 3:**
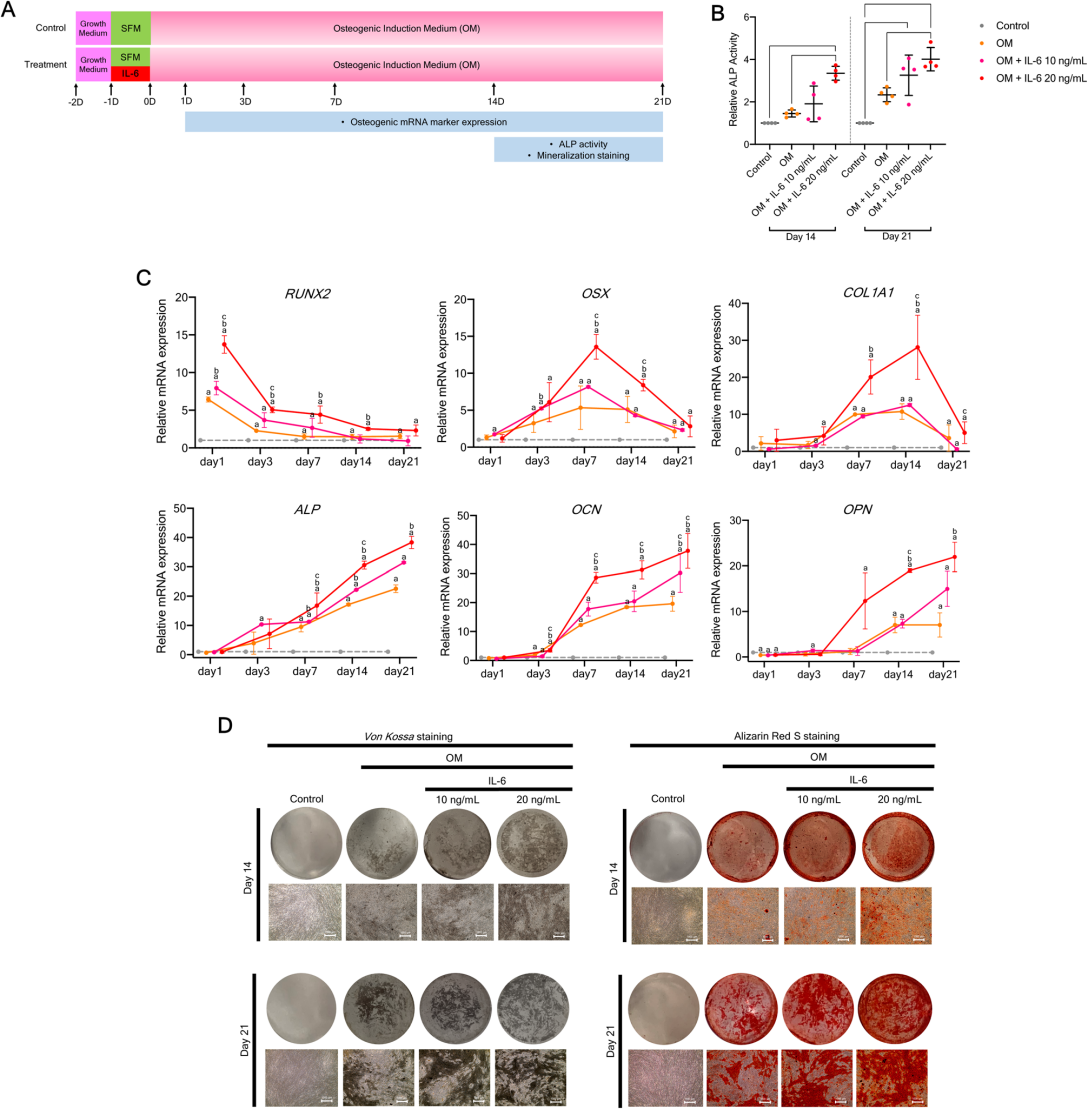
Effect of IL-6 on osteogenic differentiation potential of hPDLSCs in vitro*.* (**A**) Schematic presentation of the experiment. (**B**) Effect of IL-6 (10 and 20 ng/mL) on the in vitro osteogenic differentiation potential of hPDLSCs, as determined by ALP activity at days 14 and 21. Bars indicated significant differences (p < 0.05) (n = 4). (**C**) RT-qPCR analysis of osteogenic mRNA marker expression (*RUNX2, OSX, COL1, ALP, OCN,* and *OPN*) at days 1, 3, 7, 14, and 21 (n = 4). Superscript letters indicate significant differences vs. the undifferentiated control (^a^), osteogenic control (^b^), and osteogenic induction upon IL-6 treatment at 10 ng/mL (^c^) (*p* < 0.05). (**D**) Matrix mineralization as determined by *Von Kossa* and Alizarin Red S staining at days 14 and 21.

To assess the effect of IL-6 on osteogenic differentiation potential in vitro, hPDLSCs were pre-treated with and without IL-6 at 10 and 20 ng/mL for 24 h and then cultured in osteogenic induction medium (OM) for 21 days (Fig. 3A). The results showed that IL-6 promoted the osteogenic differentiation potential of hPDLSCs in vitro by enhancing alkaline phosphatase (ALP) activity, osteogenic mRNA marker expression, and matrix mineralization. At days 14 and 21, IL-6 at 20 ng/mL significantly enhanced the ALP activity of hPDLSCs during osteogenic induction (Fig. 3B). Additionally, IL-6 treatment significantly upregulated expression of pivotal osteogenic mRNA markers (*RUNX2, OSX, COL1, ALP, OCN,* and *OPN*) during osteogenic induction, especially at 20 ng/mL (Fig. 3C). IL-6 at 20 ng/mL also enhanced matrix mineralization of hPDLSCs as compared to the osteogenic control group, which confirmed the results of Von Kossa and Alizarin Red S staining (Fig. 3D) (n = 4). Since, IL-6 at 20 ng/mL significantly enhanced the osteogenic differentiation potential of hPDLSCs in vitro, this dosage was applied in the following experiments.

### Wnt, TGF-β1, and Notch signaling may be involved in IL-6-mediated osteogenic differentiation of hPDLSCs in vitro

**Figure 4:**
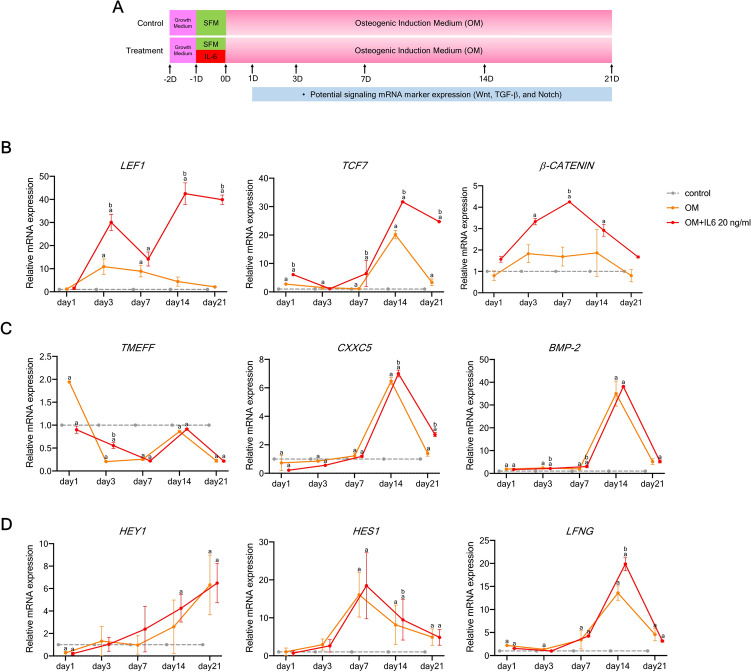
Effect of IL-6 on specific pathway target genes of hPDLSCs in vitro***.*** (**A**) Schematic presentation of the experiment. Effect of IL-6 (20 ng/mL) on specific pathway target gene expressed by hPDLSCs ((**B**) Wnt, (**C**) TGF-β1, and (**D**) Notch signaling) RT-qPCR analysis of gene expression profiles (n = 4). Superscript letters indicate significant differences vs. the undifferentiated control (^a^) and osteogenic control (^b^) (*p* < 0.05).

To further identify potential signaling pathways involved in the regulation of osteogenic differentiation, hPDLSCs pre-treated with IL-6 at 20 ng/mL for 24 h were cultured in OM for 21 days and the expression levels of specific target genes were measured on days 1, 3, 7, 14, and 21 (Fig. 4A). The results showed that the expression levels of components of the Wnt/β-catenin signaling pathway (*LEF1, TCF7*, and β-catenin) were significantly upregulated in response to IL-6 at 20 ng/mL as compared to the undifferentiated and osteogenic control groups at almost every time point (Fig. 4B), while expression of the TGF-β1 target genes (*BMP-2, TMEFF1*, and *CXXC5*) and Notch target genes (*HES1, HEY1*, and *LFNG*) was significantly increased at some, but not all, time points (Fig. 4C,D) (n = 4). The results implicated these signaling pathways in IL-6-mediated osteogenic differentiation of hPDLSCs in vitro.

### Canonical and non-canonical Wnt pathways potentially govern IL-6-mediated osteogenic differentiation of hPDLSCs in vitro

**Figure 5:**
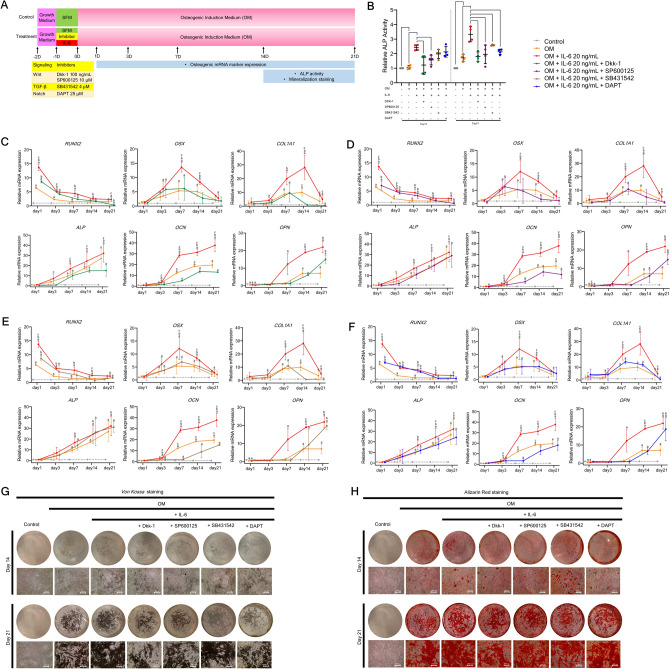
Effect of IL-6 on osteogenic differentiation potential of hPDLSCs in vitro upon the inhibition of crucial osteogenic-regulating pathways**.** (**A**) Schematic presentation of the experiment. The potential regulating pathways related to the effect of IL-6 (20 ng/mL) on osteogenic differentiation potential of hPDLSCs in vitro upon inhibition of crucial osteogenic-regulating pathways (canonical Wnt, non-canonical Wnt, TGF-β1, and Notch pathways) were investigated. Dkk-1, SP600125, SB431542, and DAPT were used to block the canonical Wnt, non-canonical Wnt, TGF-β1, and Notch signaling pathways, respectively. (**B**) ALP activity was analyzed at days 14 and 21. Bars indicated significant differences (*p* < 0.05). Osteogenic mRNA marker expression (*RUNX2, OSX, COL1, ALP, OCN,* and *OPN*) upon the inhibition of the (**C**) canonical Wnt, (**D**) non-canonical Wnt, (**E**) TGF-β1, and (**F**) Notch signaling pathways, as determined by RT-qPCR at days 1, 3, 7, 14, and 21. Superscript letters indicate significant difference vs. the undifferentiated control (^a^), osteogenic control (^b^), and osteogenic induction (with 20 ng/mL IL-6) upon specific inhibitor treatment (^c^) (*p* < 0.05). (**G**) Matrix mineralization as determined by *Von Kossa* and (**H**) Alizarin Red S staining at days 14 and 21 (n = 4).

Next, hPDLSCs pre-treated with IL-6 at 20 ng/mL were treated for 24 h with the inhibitors Dickkopf-1 (DKK1), SP600125, SB431542, and DAPT (N-[N-(3,5-difluorophenacetyl)-l-alanyl]-s-phenylglycinet-butyl ester) specific to the canonical Wnt, non-canonical Wnt, TGF-β1, and Notch pathways, respectively, and then cultured in OM for 21 days (Fig. 5A). The results showed that Dkk-1 and SP600125 significantly suppressed ALP activity at days 14 and 21, while SB431542 and DAPT suppressed ALP activity only at day 21 (Fig. 5B). Interestingly, both Dkk-1 and SP600125 dramatically downregulated expression of the pivotal bone mRNA markers *RUNX2, OSX, COL1, ALP, OCN,* and *OPN* at almost all time points, as compared to the IL-6-treated group (Fig. 5C,D), while SB431542 and DAPT significantly suppressed only some of the markers at some, but not all, time points (Fig. 5E,F). In addition, Dkk-1 and SP600125 strongly suppressed matrix mineralization at days 14 and 21, as compared to the IL-6-treated group, as determined by *Von Kossa* and Alizarin Red S staining (Fig. 5G,H) (n = 4). These results suggest that the canonical and non-canonical Wnt signaling pathways potentially govern IL-6-mediated osteogenic differentiation of hPDLSCs in vitro*.*

### *WNT2B, WNT10B,* and *WNT5A* are related to IL-6-mediated osteogenic differentiation of hPDLSCs in vitro

**Figure 6:**
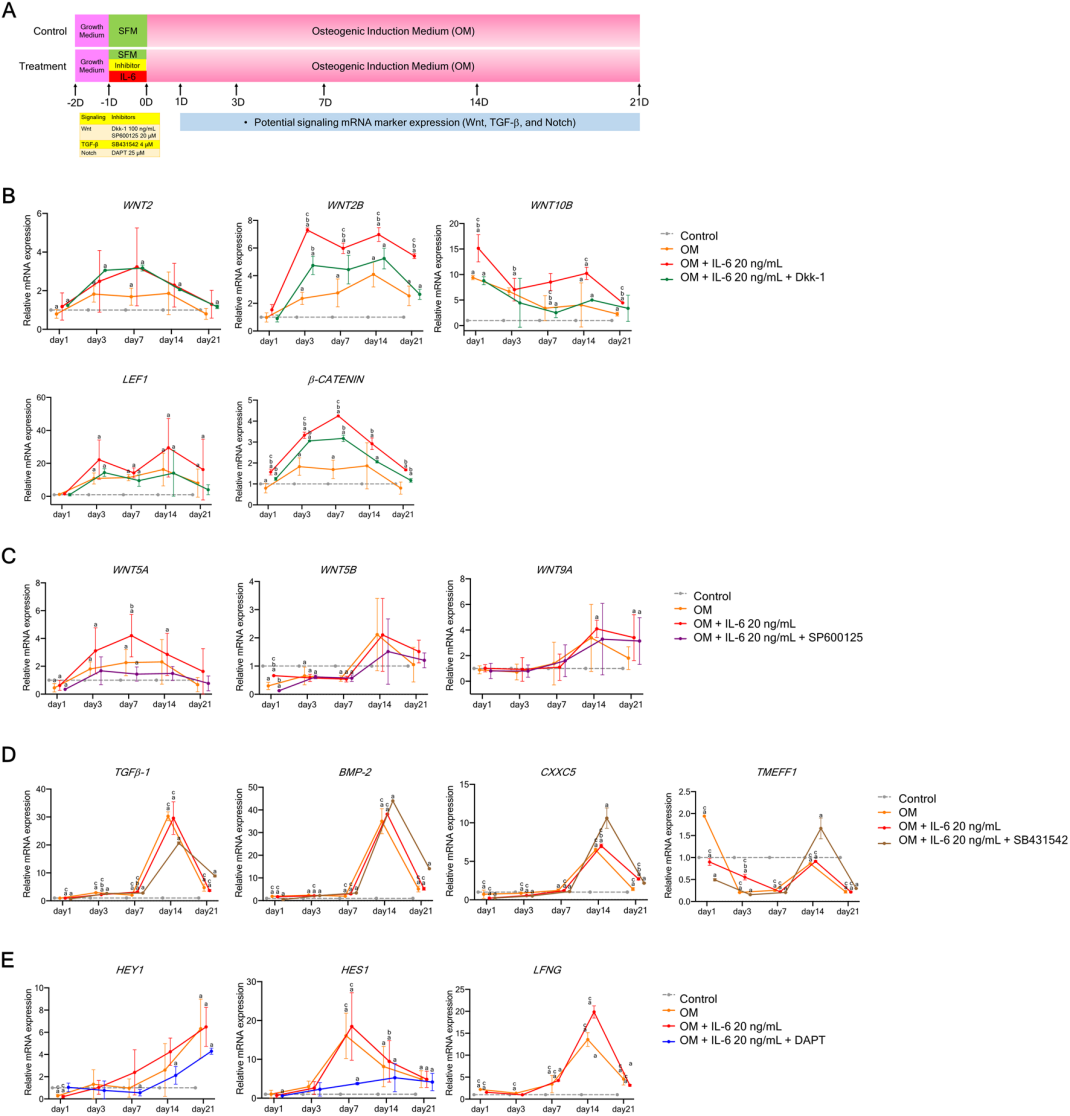
Effect of IL-6 on specific pathway target genes of hPDLSCs in vitro upon inhibition of crucial osteogenic-regulating pathways. (**A**) Schematic presentation of the experiment. To explore the relevance of potential regulating pathways, the effects of IL-6 (20 ng/mL) on specific pathway target genes expressed by hPDLSCs upon inhibition of crucial osteogenic-regulating pathways (canonical Wnt, non-canonical Wnt, TGF-β1, and Notch pathways) were analyzed. Dkk-1, SP600125, SB431542, and DAPT were used to block the canonical Wnt, non-canonical Wnt, TGF-β1, and Notch signaling pathways, respectively. Effects of IL-6 (20 ng/mL) on specific pathway target genes expressed by hPDLSCs upon treatment with each specific inhibitor of the (**B**) canonical Wnt, (**C**) non-canonical Wnt, (**D**) TGF-β1, and (**E**) Notch signaling pathways. Gene expression was quantified by RT-qPCR (n = 4). Superscript letters indicate significant differences vs. the undifferentiated control (^a^), osteogenic control (^b^), and osteogenic induction (IL-6 at 20 ng/mL) upon specific inhibitor treatment (^c^) (*p* < 0.05).

To further determine the relevance of potential signaling pathways on IL-6-mediated osteogenic differentiation, representative components of each pathway were analyzed. Thus, hPDLSCs pre-treated with IL-6 at 20 ng/mL were treated with Dkk-1, SP600125, SB431542, or DAPT for 24 h and then cultured in OM for 21 days. Afterward, the mRNA expression levels of specific ligands and transcription factors of the canonical Wnt, non-canonical Wnt, TGF-β1, and Notch pathways were measured at days 1, 3, 7, 14, and 21 (Fig. 6A). The results showed that IL-6 treatment significantly upregulated mRNA expression of some components of the canonical Wnt (*WNT2B, WNT10B, LEF1,* and *β-catenin*) and non-canonical Wnt (*WNT5A*) pathways, while the specific inhibitors Dkk-1 and SP600125 significantly suppressed expression of these markers (Fig. 6B,C). However, this trend was not observed for components of the TGF-β1 and Notch signaling pathways (Fig. 6D,E) (n = 4). These findings suggest that the canonical and non-canonical Wnt pathways participate in IL-6-mediated osteogenic differentiation of hPDLSCs in vitro via the Wnt pathway-specific ligands WNT2B, WNT10B, and WNT5A.

### WNT2B, WNT10B, and WNT5A are different key regulators on IL-6-mediated osteogenic differentiation of hPDLSCs in vitro

#### Gene silencing study

**Figure 7:**
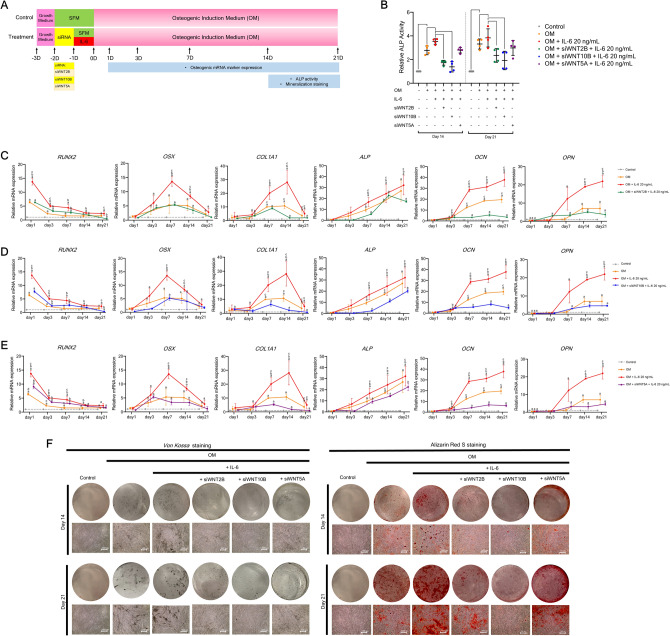
Effect of IL-6 on osteogenic differentiation potential of hPDLSCs in vitro upon silencing of WNT2B, WNT10B, or WNT5A. (**A**) Schematic presentation of the experiment. To determine the relevance of potential Wnt ligands, the effect of IL-6 (20 ng/mL) on the osteogenic differentiation potential of hPDLSCs in vitro upon silencing of WNT2B, WNT10B, or WNT5A was analyzed. siWNT2B, siWNT10B, or siWNT5A was used to knockdown the related Wnt ligand. (**B**) ALP activity was analyzed at days 14 and 21. Bars indicate significant difference (*p* < 0.05). RT-qPCR was conducted to quantify mRNA expression levels of osteogenic markers (*RUNX2, OSX, COL1, ALP, OCN,* and *OPN*) upon silencing of (**C**) WNT2B, (**D**) WNT10B, or (**E**) WNT5A at days 1, 3, 7, 14, and 21. Superscript letters indicate significant vs. the undifferentiated control (^a^), osteogenic control (^b^), and osteogenic induction (IL-6 at 20 ng/mL) upon siRNA treatment (^c^) (*p* < 0.05). (**F**) Matrix mineralization as determined by *Von Kossa* and Alizarin Red S staining at days 14 and 21 (n = 4).

To determine the relevance of WNT2B, WNT10B, or WNT5A in IL-6-mediated osteogenic differentiation of hPDLSCs, each Wnt ligand was transiently knocked down by small-interfering RNA (siRNA) upon IL-6 treatment during osteogenic induction. Then, analyses of osteogenic mRNA marker expression, ALP activity, and matrix mineralization were performed, as illustrated in Fig. 7A. Interestingly, the results showed that silencing of either WNT2B or WNT10B significantly diminished IL-6-induced ALP activity at days 14 and 21, while silencing of WNT5A only suppressed ALP activity (Fig. 7B). Further analyses showed that silencing of only WNT2B, WNT10B, or WNT5A mostly diminished IL-6-enhanced osteogenic mRNA marker expression at almost all time points (Fig. 7C–E). Moreover, silencing of each Wnt ligand clearly suppressed IL-6-enhanced matrix mineralization, as determined by *Von Kossa* and Alizarin Red S staining at days 14 and 21 (Fig. 7F) (n = 4). These results suggest that WNT2B, WNT10B, or WNT5A plays a potential role in IL-6-mediated osteogenic differentiation of hPDLSCs in vitro.

#### Recombinant WNT study

**Figure 8:**
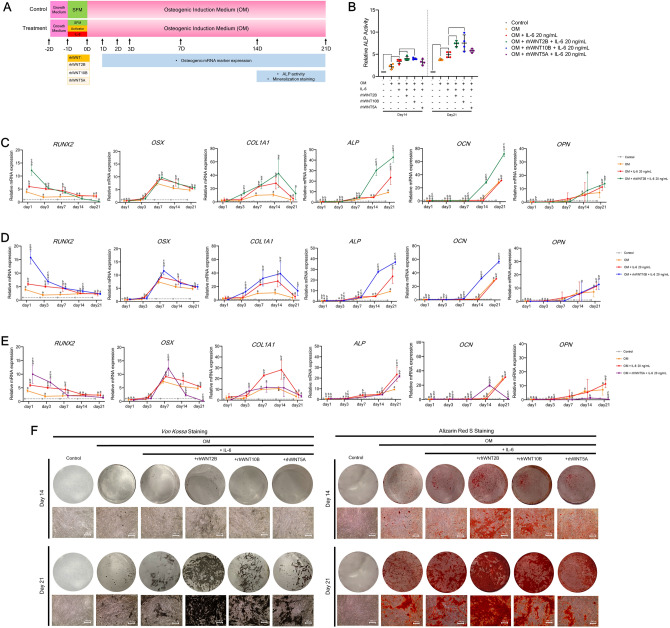
Effect of IL-6 on the osteogenic differentiation potential of hPDLSCs in vitro upon treatment with rhWNT2B, rhWNT10B, or rhWNT5A. (**A**) Schematic presentation of the experiment. To confirm the relevance of potential Wnt ligands, the effect of IL-6 (20 ng/mL) on the osteogenic differentiation potential of hPDLSCs in vitro upon treatment with rhWNT2B, rhWNT10B, or rhWNT5A was analyzed. rhWNT was used to mimic each Wnt ligand. (**B**) ALP activity was analyzed at days 14 and 21. Bars indicate significant differences (*p* < 0.05). RT-qPCR analysis of mRNA expression of osteogenic markers (*RUNX2, OSX, COL1, ALP, OCN,* and *OPN*) upon treatment with (**C**) rhWNT2B, (**D**) rhWNT10B or (**E**) rhWNT5A at days 1, 3, 7, 14, and 21. Superscript letters indicate significant difference vs. the undifferentiated control (^a^), osteogenic control (^b^), and osteogenic induction (with 20 ng/mL IL-6) upon rhWNT treatment (^c^) (*p* < 0.05). **f**, Matrix mineralization as determined by *Von Kossa* and Alizarin Red S staining at days 14 and 21 (n = 4).

To further confirm the relevance of WNT2B, WNT10B, or WNT5A on IL-6-mediated osteogenic differentiation of hPDLSCs, each recombinant human WNT (rhWNT) was applied upon IL-6 treatment during osteogenic induction. Then, analyses of osteogenic mRNA marker expression, ALP activity, and matrix mineralization were performed as illustrated in Fig. 8A. The results showed that either rhWNT2B or rhWNT10B significantly enhanced IL-6-mediated ALP activity at days 14 and 21 (Fig. 8B). Further analyses revealed that either rhWNT2B or rhWNT10B significantly enhanced IL-6-mediated upregulation of pivotal osteogenic mRNA markers (*RUNX2, COL1, ALP,* and *OCN*) (Fig. 8C,D), while rhWNT5A upregulated only some osteogenic mRNA markers (*RUNX2* and *OCN*), as compared to the IL-6-treated group (Fig. 8E). Interestingly, either rhWNT2B or rhWNT10B obviously enhanced IL-6-mediated matrix mineralization, as determined by *Von Kossa* and Alizarin Red S staining at day 21, while the effect of rhWNT5A was comparatively diminished (Fig. 8F) (n = 4). The results of the rhWNT study suggest that either WNT2B or WNT10B plays a potential role in IL-6-mediated osteogenic differentiation of hPDLSCs in vitro*,* while WNT5A may have a comparatively lesser impact.

#### Stabilization and translocation of β-catenin study

**Figure 9:**
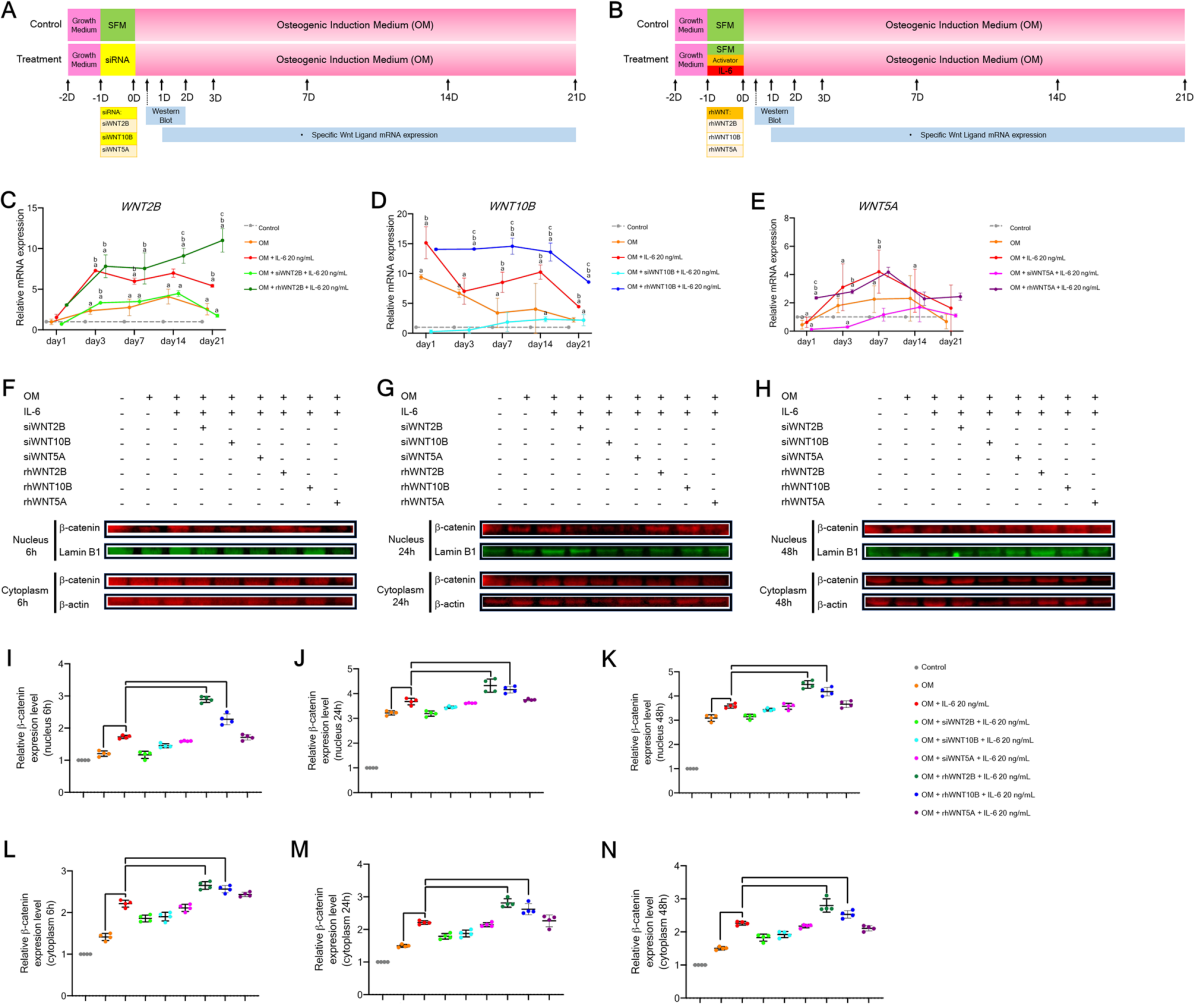
β-Catenin stabilization and translocation. (**A**,**B**) Schematic presentation of the experiment using siRNAs and rhWNTs. To explore the dynamic of crucial Wnt ligands and the relevance of Wnt/β-catenin-dependent pathway, the effect of IL-6 (20 ng/mL) on expression of *WNT2B, WNT10B,* and *WNT5A* along with stabilization and translocation of β-catenin in hPDLSCs during in vitro osteogenic induction upon treatment with siRNAs or rhWNTs was analyzed. Sets of siRNAs (siWNT2B, siWNT10B, or siWNT5A) and rhWNTs (rhWNT2B, rhWNT10B, or rhWNT5A) were used. RT-qPCR analysis of the mRNA expression levels of crucial Wnt ligands, including (**C**) *WNT2B,* (**D**) *WNT10B,* and (**E**) *WNT5A*, at days 1, 3, 7, 14, and 21. Superscript letters indicate significant difference vs. the undifferentiated control (^a^), osteogenic control (^b^), and osteogenic induction (IL-6 at 20 ng/mL) (^c^) (*p* < 0.05). Subcellular stabilization and translocation of β-catenin at (**F**) 6, (**G**) 24, and (**H**) 48 h, respectively. Quantitative expression of β-catenin in the nuclear fraction at (**I**) 6, (**J**) 24, and (**K**) 48 h along with expression in the cytoplasmic fraction at (**L**) 6, (**M**) 24, and (**N**) 48 h, as determine with the QuanTL program. Lamin B1 and β-ACTIN were used as reference proteins in the nuclear and cytoplasmic fractions, respectively (n = 4). Bars indicate significant difference (*p* < 0.05). Original blots are presented in Supplementary Figs. 1A–3C.

**Figure 10:**
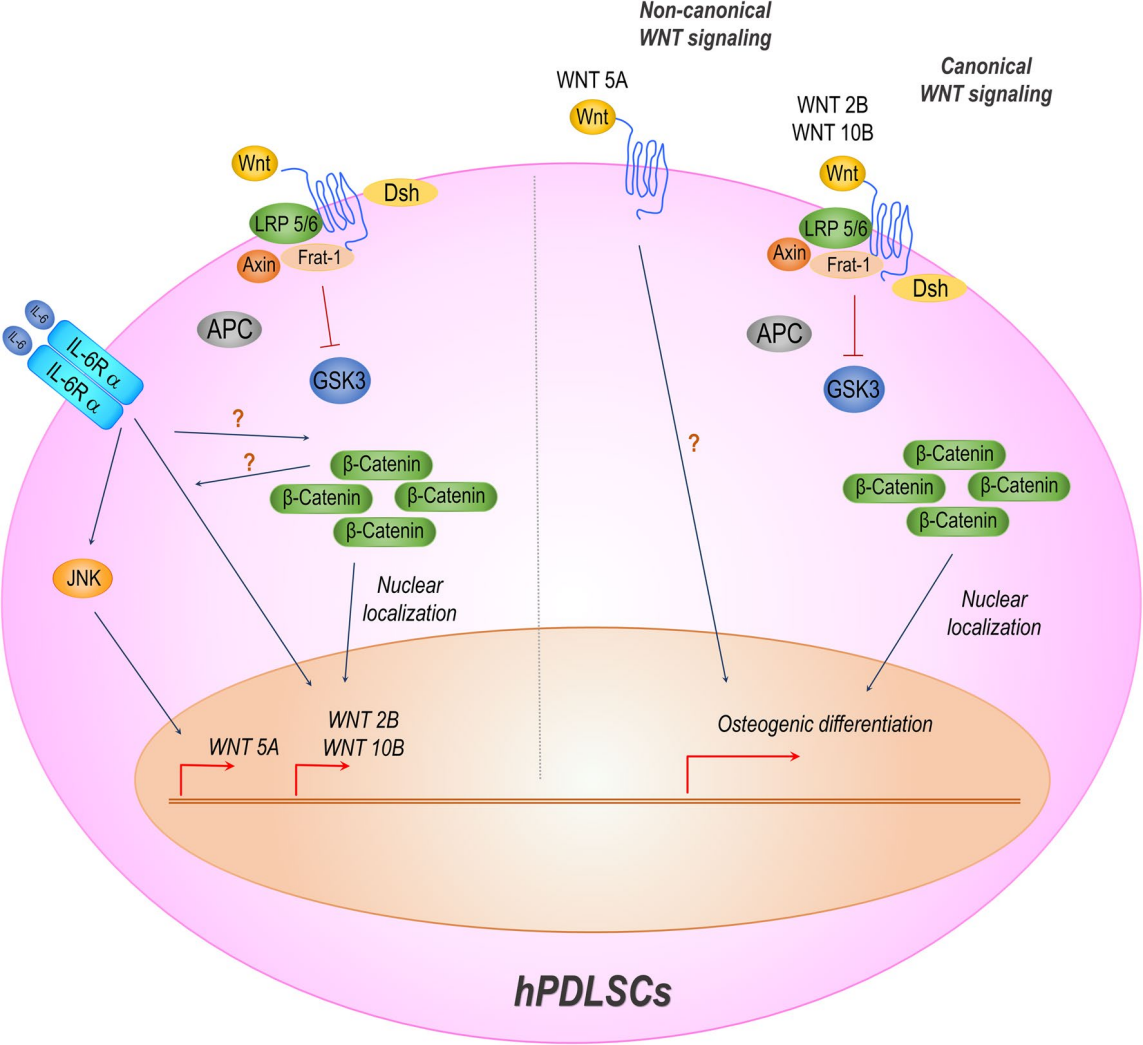
Summary of Wnt-related signaling pathways governing osteogenic differentiation of hPDLSCs treated with IL-6. The infographic illustrates potential Wnt-related signaling pathways involved in osteogenic differentiation of hPDLSCs treated with IL-6 in vitro*.* IL-6 employed the canonical Wnt/β-catenin pathway via either WNT2B or WNT10B to benefit the osteogenic differentiation potential of hPDLSCs in vitro. Besides, IL-6 may rely on the non-canonical Wnt pathway via WNT5A to promote osteogenic differentiation of hPDLSCs in vitro.

To explore the roles of *WNT2B, WNT10B,* and *WNT5A* along with the relevance of the Wnt/β-catenin-dependent pathway in IL-6-mediated osteogenic differentiation of hPDLSCs, either siRNA or rhWNT of each Wnt ligand was applied upon IL-6 treatment during osteogenic induction. Then, mRNA expression of each Wnt ligand and β-catenin stabilization and translocation were analyzed (Fig. 9A,B). The results showed that siRNA significantly downregulated expression of *WNT2B, WNT10B,* and *WNT5A* at all time points, as compared to the IL-6-treated group, while rhWNT significant upregulated *WNT2B* and *WNT10B* at almost all time points. However, as compared to the IL-6 treated group, *WNT5A* was significantly upregulation only at day 1 (Fig. 9C–E). These results illustrate the dynamic expression of *WNT2B, WNT10B,* and *WNT5A* upon IL-6 treatment. Further analyses of subcellular β-catenin stabilization and translocation showed that, upon stimulation by rhWNT, both rhWNT2B and rhWNT10B, but not rhWNT5A, significantly enhanced cellular stabilization and nuclear translocation of β-catenin at all time points (6, 24, and 48 h) (Fig. 9F–N, Supplementary Figs. 1A–3B) (n = 4), suggesting that IL-6 employed the canonical Wnt/β-catenin-dependent pathway via either WNT2B or WNT10B to promote the osteogenic differentiation potential of hPDLSCs in vitro. Besides, IL-6 may also employ the non-canonical Wnt pathway via WNT5A. A summary of the Wnt-related signaling pathways governing osteogenic differentiation of hPDLSCs treated with IL-6 is presented in Fig. 10.

## Discussion

Homeostasis of alveolar bone and periodontal tissue plays an important role in the progression of oral and periodontal diseases^12,48^. The most important factor influencing homeostasis of these tissues is the state of inflammation^17^. Chronic inflammation that accompanies severe periodontitis can lead to destruction of periodontal tissue and alveolar bone^3^. The inflammatory response within the tissue is dependent on the balance of pro- and anti-inflammatory cytokines^21,23^. In the early stage of inflammation, IL-6 release is promoted by various pro-inflammatory cytokines, including IL-1, IL-3, tumor necrosis factor alpha (TNF-α), interferon gamma, and platelet-derived growth factor^17,18^. Previous studies have reported that IL-1 and TNF-α are associated with bone loss in oral diseases^19,49^. IL-1 stimulates T and B cells to amplify inflammatory responses and promotes macrophage production of receptor activator nuclear factor-kappa B (RANK) and the corresponding ligand RANKL. Binding of RANKL to RANK promotes monocyte/macrophage differentiation toward preosteoclasts, which leads to osteoclastogenesis and the process of bone resorption^17,18^. In addition, TNF-α increases osteoclastic bone resorption and inhibits osteoblast differentiation^19^.

On the other hand, IL-6 plays a biphasic role in bone tissue homeostasis^50^: inhibition of osteoclastogenesis^51,52^ and initiation of osteolysis and osteoporosis^53,54^. Expression of IL-6 is upregulated in inflamed periodontal tissues^55,56^. Interestingly, expression of IL-6 has also been detected in other dental tissues and cells^57,58^, suggesting that IL-6 is closely related to the homeostatic capacity of oral and periodontal tissues. Therefore, comprehensive understanding of the role of IL-6 is key to address the destruction of alveolar bone and periodontal tissue caused by inflammation associate with periodontal tissues.

In this study, hPDLSCs were used to clarify the effects of IL-6 in periodontal tissue, especially osteogenic differentiation potential. Furthermore, a comprehensive study was conducted to elucidate the possible underlying mechanisms. Previous reports have shown that IL-6 at 10 ng/mL could benefit the proliferative potential of tendon-derived stem cells^15^, while IL-6 at 20 ng/mL enhanced osteogenic differentiation of human bone marrow stromal cells (hBMSCs)^20^. Preliminarily observations of the effects of IL-6 at 10 and 20 ng/mL on the proliferation of hPDLSCs found that IL-6 at both dosages was not cytotoxic and did not influence the proliferative capacity of viability of cells. A previous study found that IL-6 had no effect on the proliferative capacity of hBMSCs or mRNA expression levels of the cell cycle regulators cyclin *D1, E1,* and *B1*^20^. The results of the present study confirmed that IL-6 does not significantly affect the proliferation and viability of hPDLSCs. However, IL-6 at 20 ng/mL significantly benefited the osteogenic differentiation potential of hPDLSCs, as demonstrated by increased ALP activity, osteogenic mRNA expression, and matrix mineralization, while the effects of IL-6 at 10 ng/mL were relatively lower. These findings are in agreement with those of previous studies showing the positive effects of IL-6 on the osteogenic differentiation potential of hBMSCs^20^, hBM-MSCs^41^, and human adipose stem cells^59^. In addition, IL-6 administration with recombinant human bone morphogenetic protein-2 and absorbable collagen sponge implantation enhanced ectopic bone formation in a rat model, although extensive adipogenic differentiation also occurred^20^. In a previous study, treatment of stem cells isolated from human exfoliated deciduous teeth (SHEDs) with IL-6 at 10 ng/mL enhanced stemness mRNA marker expression and osteogenic differentiation without influencing the capacity for adipogenic and neurogenic differentiation^25^. This finding suggests that IL-6 promotes osteogenic differentiation of various MSCs, depending on the cell type and study model.

To achieve desirable therapeutic outcomes for oral and periodontal diseases, the treatment regimen should not only prevent disease progression, but also enhance tissue reconstruction and regeneration^37,38^. Accordingly, advanced therapeutic regimens, such as stem cell-based bone tissue engineering, are widely used for reconstruction of oral and periodontal tissues^11,60–63^. As a potential cell resource, hPDLSCs can promote osteogenesis and reconstruction of periodontal tissue or implantation^64^ due to excellent osteogenic differentiation potential and bone matrix production^25,65^. According to the International Society for Cellular Therapy^66^, hPDLSCs and MSCs share some common characteristics, as described in our previous reports^30,32^. The hPDLSCs in the present study were characterized by spindle-shaped morphology, mRNA markers of stemness and proliferative capacity, MSC-related surface markers, colony-forming capacity, and the potential to differentiate into osteogenic, adipogenic, and chondrogenic lineages. Hence, owing to the efficiency of the isolation protocol and robustness of the isolated cells, hPDLSCs are a good candidate for stem cell-based bone tissue engineering. Notably, IL-6 is reported to enhance the stemness of both SHEDs and BM-MSCs, but by different mechanisms^26,40,67,68^. Therefore, further studies on the effects of IL-6 on the stemness of hPDLSCs and the underlying mechanisms are warranted.

The present study is the first to report and confirm that IL-6 employed the canonical Wnt/β-catenin-dependent pathway via either WNT2B or WNT10B to promote osteogenic differentiation of hPDLSCs in vitro. Alternatively, IL-6 may also employ the non-canonical Wnt pathway via WNT5A. Three analytic approaches were used to confirm these findings: gene silencing, recombinant Wnt, and β-catenin stabilization/translocation. However, the mechanisms underlying osteogenic differentiation of MSCs vary among different species and tissue origins^32,69–75^, which may explain the various signaling pathways employed by IL-6 for osteogenic differentiation and stemness properties^24–26,76–78^. Furthermore, a previous study reported that IL-6 employed the extracellular signal-regulated kinase (ERK)1/2-dependent pathway to maintain the stemness of hBM-MSCs in vitro^40^, while another found that IL-6 downregulated SRY-box transcription factor 2 expression, which subsequently impaired the multipotency of hBM-MSCs with commitment toward the osteogenic lineage, as demonstrated by upregulation of osteogenic mRNA markers^68^. Additional evidence suggests that osteogenic differentiation of hBM-MSCs is, at least partially, regulated by IL-6 and the membranous IL-6 receptor (IL-6R) via activation of signal transducer and activator of transcription 3^41^. MC3T3-E1 cells seeded on hydroxyapatite showed that the combination of IL-17A and IL-6 enhanced osteogenic differentiation by increasing the osteoprotegerin/RANKL ratio^79^.

Interestingly, the relationship between IL-6 and osteogenic differentiation of MSCs via Wnt signaling has not yet been elucidated. The results of the present study showed that the canonical and non-canonical Wnt pathways were employed for osteogenic differentiation of hPDLSCs treated with IL-6 via WNT2B or WNT10B and WNT5A. A previous report mentioned that the H_2_S-donor GYY4137 enhanced murine osteoblastogenesis via Wnt signaling, as demonstrated by increased expression of Wnt16, Wnt2b, Wnt6, and Wnt10b^80^. A study of chronic arthritis of the rat temporomandibular joint found that Wnt10b expression was increased in the synovial membrane with subsequent expression of Dkk-1 via a negative feedback loop^80^. An additional report of hPDLSCs isolated from inflamed tissues found that microRNA-26a-5p enhanced osteogenic differentiation by targeting Wnt5a expression via a related downstream pathway^81^. In regard to matrix production, Wnt5a was shown to induce collagen production by hPDLSCs via periostin expression through the TGF-β1 pathway^82^.

Since the Wnt signaling plays an important role in oral and maxillofacial diseases^83^, further clarification of the mechanisms underlying the activities of IL-6 in osteogenic differentiation of hPDLSCs is especially important for management of oral and periodontal diseases as well as tissue regeneration.

## Materials and methods

### Study approval and patient consent

The study protocol was approved by the Human Research Ethics Committee, Faculty of Dentistry, Chulalongkorn University (approval code: HREC-DCU 2018/054) and conducted in accordance with the ethical principles for medical research involving human subjects described in the Declaration of Helsinki. Prior to inclusion in this study, written informed consent was obtained from all subjects.

### Cell isolation, culture, and expansion

The hPDLSCs used in this study were obtained from the healthy extracted wisdom tooth of female and male subjects, aged 18–35 years, who visited the clinic for non-orthodontic reasons. The isolation protocol was modified from previous reports^25,32,84,85^. The hPDLSCs were isolated via the tissue explant technique. Briefly, human periodontal ligament tissues were collected from the mid tooth root, placed in a 35-mm culture dish, and maintained in culture medium at 37 °C under an atmosphere of 5% CO_2_. At 80% confluence, the cells were subcultured and transferred to a 60-mm culture dish. The culture medium was changed every 48 h and the cells were subcultured to 80% confluence in Dulbecco’s modified Eagle’s medium (Thermo Fisher Scientific) supplemented with 10% fetal bovine serum (Thermo Fisher Scientific), 1% Gibco™ Antibiotic–Antimycotic (Thermo Fisher Scientific), and 1% Glutamax (Thermo Fisher Scientific). Cells at passage 2–5 were used for the experiments.

Recombinant human IL-6 (cat. no. PHP045; Bio-Rad Laboratories, Hercules, CA, USA) was used at concentrations of 10 and 20 ng/mL^15,20^.

### Cell characterization

The isolated hPDLSCs were characterized in accordance with the International Society for Cell Therapy guidelines^66^. Flow cytometry was employed to assess the MSC surface markers CD105, CD73, CD90, and CD44, and the hematopoietic cell surface marker CD45. Briefly, single-cell suspensions of hPDLSCs (1.7 × 10^6^ cells/mL) were stained with fluorescein isothiocyanate (FITC)-conjugated anti-human CD105 antibody (BioLegend, San Diego, CA, USA), FITC-conjugated anti-human CD73 antibody (BioLegend), FITC-conjugated anti-human CD90 antibody (BioLegend), FITC-conjugated anti-human CD44 antibody (BioLegend), or FITC-conjugated anti-human CD45 antibody (BioLegend). FITC-conjugated Mouse IgG1κ Isotype (BioLegend) was used as isotype control. Prior to staining, the cells were incubated for 1 h. The results were analyzed using a FACSCalibur™ flow cytometer (BD Biosciences, San Jose, CA, USA) and CellQuest™ software (BD Biosciences).

Quantitative reverse transcription polymerase chain reaction (RT-qPCR) was performed to measure the mRNA expression levels of the stemness markers *REX-1*, *NANOG*, and *OCT-4*, and the cell proliferation marker *Ki67*.

The differentiation potential of hPDLSCs toward the osteogenic, adipogenic, and chondrogenic lineages was investigated in accordance with published protocols with slight modifications^32,69,86,87^. For osteogenic differentiation, cells were seeded into the wells of 24-well culture plates at 3 × 10^4^ cells/well. After 24 h, cells were maintained for 21 days in OM supplemented with 50 mg/mL of ascorbic acid (Sigma-Aldrich Corporation, St. Louis, MO, USA), 100 nM dexamethasone (Sigma-Aldrich Corporation), and 10 mM β-glycerophosphate (Sigma-Aldrich Corporation). Osteogenic differentiation potential was assessed by Von Kossa and Alizarin Red staining for mineralization of the extracellular matrix. The mRNA expression levels of the osteogenic markers *RUNX2, OSX, COL1A1, ALP, OCN,* and *OPN* were determined by RT-qPCR. Undifferentiated cells were used as a control.

For adipogenic differentiation, cells were seeded in to the wells of 24-well culture plates (5 × 10^4^ cells/well) and maintained in adipogenic induction medium containing 1 µM dexamethasone (Sigma-Aldrich Corporation), 0.1 mM indomethacin (Sigma-Aldrich Corporation), 1 mM 3-isobutyl-1-methylxanthine (Sigma-Aldrich Corporation), and 0.1 mg/mL of insulin (Sigma-Aldrich Corporation) for 72 h, followed by adipogenic maintenance medium containing 0.1 mg/mL of insulin for 24 h. The induction cycle was repeated four times. Cells were then cultured in adipogenic maintenance medium until day 28. To analyze the differentiation potential, cells were stained for 1 h with Oil Red O solution (Sigma-Aldrich Corporation). Intracellular lipid droplets were observed under a microscope. The mRNA expression levels of the adipogenic mRNA markers *LPL* and *PPRγ* were measured by RT-qPCR.

For chondrogenic differentiation, cells were seeded into the wells of 24-well culture plates (3 × 10^4^ cells/well) and maintained for 21 days in chondrogenic induction medium supplemented with 1% l-glutamine (Thermo Fisher Scientific, Waltham, MA, USA), 1% Gibco™ Antibiotic–Antimycotic (Thermo Fisher Scientific), 50 mg/mL of ascorbic acid (Sigma-Aldrich Corporation), 40 mg/mL of l-proline (Sigma-Aldrich Corporation), 0.1 µM dexamethasone, 1% insulin-transferrin-selenium (Thermo Fisher Scientific), 10 ng/mL of TGF-β3 (Sigma-Aldrich Corporation), and 15% fetal bovine serum. Chondrogenic differentiation was analyzed by Alcian Blue staining of glycosaminoglycans. The expression levels of the chondrogenic mRNA markers *SOX9* and *COL10* were determined by RT-qPCR.

### RT-qPCR

Total cellular RNA was isolated using TRIzol^®^ reagent (Invitrogen Corporation, Carlsbad, CA, USA) in accordance with the manufacturer’s protocol. The mRNA content was isolated using the Direct-zol™ RNA Miniprep kit (ZymoResearch, USA). The quantity of the extracted RNA was determined using the Qubit™ RNA High Sensitivity Broad Range Assay kit (Thermo Fisher Scientific). Complementary DNA was reverse transcribed from 1 µg of mRNA using the ImProm-II™ Reverse Transcription System kit (Promega Corporation, Madison, WI, USA). RT-qPCR was performed using PowerUp™ SYBR™ Green Master Mix (Thermo Fisher Scientific), the primer sequences listed in Table 1, and a Bio-Rad Real-Time PCR Detection System (Bio-Rad Laboratories). The final mRNA expression values were normalized to the 18S ribosomal RNA gene according to the following formula 2^−ΔΔCt^, where ΔΔCt = [Ct_target gene_ − Ct_*18S*_] treatment group − [Ct_target gene_ − Ct_*18S*_] control group.

**Table 1:** Primer sequences.

No.	Gene	Accession number	Forward primer sequence	Reverse primer sequence
1	*18S*	NR_046235.3	GTGATGCCCTTAGATGTCC	CCATCCAATCGGTAGTAGC
2	*REX1*	NM_174900.5	TGGGAAAGCGTTCGTTGAGA	CACCCTTCAAAAGTGCACCG
3	*NANOG*	NM_024865.4	ATGCCTCACACGGAGACTGT	AAGTGGGTTGTTTGCCTTTG
4	*OCT-4*	NM_002701.6	TCGAGAACCGAGTGAGAGG	GAACCACACTCGGACCACA
5	*Ki67*	NM_001145966.1	TCAGAATGGAAGGAAGTCAACTG	TCACTCTCATCAGGGTCAGAAG
6	*LEF1*	XM_005263046.3	TCTTCCTTGGTGAACGAGTCT	GATGCTTTCCGTCATCGGG
7	CTNNB1	NM_001098209.1	ATGGCTTGGAATGAGACTGCT	GGGTCCATACCCAAGGCATC
8	*WNT2*	NM_003391.2	TCTCGGTGGAATCTGGCTCT	GGCACATTATCGCACATCACC
9	*WNT2B*	NM_001291880.1	CCGAGAGTGTCAGCACCAAT	TGGACTACCCCTGCTGATGA
10	*WNT5A*	NM_003392.7	TCAGGCACCATTAAACCACA	AATTCACAGAGGTGTTGCAGC
11	*WNT5B*	XM_024449207.1	AAGGAGGACGCGTAGCAAGA	TGACAGTTTCCAGAGTAGGGTTC
12	*WNT9A*	NM_003395.3	TTCCACAACAACCTCGTGGG	TTCAGATGCTTGCCCACCTC
13	*WNT10B*	NM_003394.4	ATCCTCAAGCGCGGTTTCC	AACTCTTGCCTCGGGACAGT
14	*TMEFF1*	NM_003692.5	TGCTTTCTCAGAAGGGCTGC	CCTGACCCTTCCTCTTCTCCT
15	*CXXC5*	NM_001317199.2	GGCAAGAAGAAGCGGAAACG	TCGGAAGCATCACCTTCTCC
16	*BMP-2*	NM_001200.4	TGCGGTCTCCTAAAGGTCG	AACTCGAACTCGCTCAGGAC
17	*TGF-β1*	NM_000660.7	GGATACCAACTATTGCTTCAGCT	AGGCTCCAAATGTAGGGGCAGGG
18	*HEY1*	NM_012258.3	TAATTGAGAAGCGCCGACGA	GCAACTTCTGCCAGGCATTC
19	*HES1*	NM_005524.3	AGGCGGACATTCTGGAAATG	CGGTACTTCCCCAGCACACTT
20	*LFNG*	NM_001040167.2	GATCTCGCGCCACAAGGAG	ACGTGGCAGAACCACTTCC
21	*RUNX2*	NM_001024630.4	CCCCACGACAACCGCACCAT	CACTCCGGCCCACAAATC
22	*OSX*	NM_001173467.3	GCCAGAAGCTGTGAAACCTC	GCTGCAAGCTCTCCATAACC
23	*COL1A1*	NM_000088.4	CTGGCAAAGAAGGCGGCAAA	CTCACCACGATCACCACTCT
24	*ALP*	NM_000478.6	CGAGATACAAGCACTCCCACTTC	CTGTTCAGCTCGTACTGCATGTC
25	*OCN*	NM_199173.6	CTTTGTGTCCAAGCAGGAGG	CTGAAAGCCGATGTGGTCAG
26	*OPN*	NM_001040058.2	AGGAGGAGGCAGAGCACA	CTGGTATGGCACAGGTGATG
27	*PPARG*	NM_001330615.4	GTGACCAGAAGCCTGCATTT	GTCAACCATGGTCATTTCGTT
28	*LPL*	NM_000237.3	CATGGCTGGACGGTAACAGG	CGGACACTGGGTAATGCTCC
29	*SOX9*	NM_000346.4	GGCAAGCTCTGGAGACTTCTG	CCCGTTCTTCACCGACTTCC
30	*COL10A1*	NM_000493.4	TCCCAGCACGCAGAATCCATC	TGTCTTGGTGTTGGGTAGTGG

*18S* 18S ribosomal ribonucleic acid, *REX1* reduced expression 1, *NANOG* NANOG homeobox, *OCT-4* octamer transcription factor 4, *KI67* Kiel original clone-67, *LEF1* lymphoid enhancer binding factor 1, *CTNNB1* catenin beta1, *WNT2* wingless family member 2, *WNT2B* wingless family member 2B, *WNT5A* wingless family member 5A, *WNT5B* wingless family member 5B, *WNT9A* wingless family member 9A, *WNT10B* wingless family member 10B, *TMEFF1* tomoregulin 1, *CXXC5* CXXC finger protein 5, *BMP2* bone morphogenetic protein, *TGF-β1* transforming growth factor beta1, *HEY1* Hes related with YPRW motif protein, *HES1* hairy and enhancer of split-1, *LFNG* lunatic fringe, *RUNX2* Runt-related transcription factor 2, *OSX* osterix, *COL1A1* collagen type I alpha 1 chain, *ALP* alkaline phosphatase, *OCN* osteocalcin, *OPN* osteopontin, *PPARG* peroxisome proliferator activated receptor gamma, *LPL* lipoprotein lipase, *SOX9* SRY-box transcription factor 9, and *COL10A1* collagen type X alpha 1 chain.

### Colony-forming assay

In accordance with a previous published protocol, hPDLSCs (500 cells per dish) were cultured in a 60-mm culture dish (Corning Incorporated, Corning, NY, USA) for 14 days^32,88^, stained with crystal violet (Sigma-Aldrich Corporation), as describe elsewhere^32,69^, washed with phosphate-buffered saline (PBS), and fixed with 100% methanol (Sigma-Aldrich Corporation) for 20 min at 4 °C. Colonies containing more than 50 aggregated cells were counted.

### Proliferation and live/dead assays

Cell proliferation was evaluated using the alamarBlue™ assay. Briefly, cells were incubated in culture medium supplemented with 5% alamarBlue™ (Invitrogen Corporation) for 3 h. The spectrophotometric absorbance of each sample was recorded at wavelengths of 570 and 600 nm for reduced and oxidized spectrums, respectively. The percentage of reduction was calculated in accordance with the manufacturer’s protocol.

Cell viability was assessed by staining with calcein-AM (live cells—green fluorescence) and propidium iodide (dead cells—red fluorescence) and evaluated with a fluorescent microscope (ApoTome.2; Carl Zeiss Microscopy, LLC, Thornwood, NY, USA).

### ALP activity

ALP activity was observed at days 14 and 21 after osteogenic induction, as described previously^32,69,70^. hPDLSCs were lysed in lysis buffer containing 0.1% Triton X-100, 1 M Tris–HCl, and 5 mM MgCl_2_. Lysates were incubated with *p*-nitrophenol phosphate (Life Technologies, Carlsbad, CA, USA), 2-amino-2-methyl-1-propanolol (Sigma-Aldrich Corporation), and 2 mM MgCl_2_ for 15 min at 37 °C. The reactions were terminated by the addition of 0.1 M NaOH. Then, absorbance was measured at a wavelength of 410 nm. The total protein concentration was measured with a Qubit™ Protein Assay kit (Thermo Fisher Scientific). ALP activity is presented as U/mg of protein.

### Mineralization assay

*Von Kossa* and Alizarin Red staining was used to examine mineral deposition at days 14, and 21 after osteogenic induction, as described in a previous report^69,86^. For *Von Kossa* staining, hPDLSCs were washed with PBS, fixed with cold methanol at 4 °C for 15 min, washed with deionized (DI) water, and incubated with 5% silver nitrate solution (Sigma-Aldrich Corporation) under ultraviolet light for 30 min. Afterward, cells were washed with DI water and then mixed with 5% sodium thiosulfate (Sigma-Aldrich Corporation) for 5 min at room temperature (RT) to remove unreacted silver. Sodium thiosulfate was rinsed with DI water. Phosphate-deposited mineralization was observed under an inverted microscope as dark brown-black nodules.

For Alizarin Red S staining, hPDLSCs were washed with PBS, fixed with cold methanol for 15 min at 4 °C, washed three times with DI water (pH 4.2), and stained with 2% Alizarin Red S solution (Sigma-Aldrich Corporation) for 5 min at RT. Afterward, cells were washed 2–3 times with DI water (pH 4.2) to remove excess stain. Calcium-deposited mineralization was observed as a red color under an inverted microscope.

### Signaling pathway blocking experiments

hPDLSCs were treated with specific inhibitors for 24 h, as described in previous studies^24,32,69,89–91^. The canonical Wnt, non-canonical Wnt, TGF-β1, and Notch signaling pathways were inhibited with recombinant human Dkk-1 at 100 ng/mL (R&D Systems, Inc., Minneapolis, MN, USA), SP600125 at 10 µM (Sigma-Aldrich Corporation), SB431542 at 4 µM (Sigma-Aldrich Corporation), and DAPT at 20 µM (Sigma-Aldrich Corporation), respectively.

### siRNA and rhWnt experiments

For gene knockdown, hPDLSCs were treated with *siWNT2B*, s*iWNT10B*, and *siWNT5A* (Ambion, Foster City, CA, USA). To prepare the transfection medium, siRNA at 10 pmol/μL and oligonucleotide at 5 nM were added to 3 μL of Lipofectamine RNAi-MAX transfection reagent (Thermo Fisher Scientific). Then, the mixture was added to 500 μL of Opti-MEM reduced serum medium. hPDLSCs (3 × 10^4^) were seeded into the wells of 24-well plate and maintained in transfection medium for 24 h.

For rhWNT2B (Cusabio Biotech Co., Ltd., Wuhan, China), rhWNT10B, and rhWNT5A (R&D Systems, Inc.) treatment, the rhWNT medium contained 50 ng/mL of rhWNT ligand in serum-free medium. hPDLSCs (3 × 10^4^) were seeded into the wells of a 24-well plate and cultured in rhWNT medium for 24 h.

### Protein extraction by subcellular fractionation

Cytoplasmic and nuclear protein fractionation was performed in accordance with an established subcellular fractionation protocol. hPDLSCs were rinsed in cold 1 × PBS and trypsinized at 6, 12, and 24 h post-induction. All samples were lysed with subcellular fractionation buffer containing HEPES (4-(2-hydroxyethyl)-1-piperazineethanesulfonic acid) (Thermo Fisher Scientific), KCl (Sigma-Aldrich Corporation), MgCl_2_ (Sigma-Aldrich Corporation), ethylenediaminetetraacetic acid (Sigma-Aldrich Corporation), egtazic acid (Sigma-Aldrich Corporation), 1 mM dithiothreitol (Affymetrix, Santa Clara, CA, USA), protease, and phosphatase inhibitor (Thermo Fisher Scientific). After centrifugation, the nuclear pellet was collected and homogenized with an ultrasonic probe sonicator (PRO Scientific, Inc., Oxford, CT, USA). The supernatant was then separated by ultracentrifugation (Beckman Coulter, Inc., Brea, CA, USA) at 300,000 RCF to obtain fractions of cytoplasmic proteins.

### Western blot analysis

Protein concentrations were measured using the bicinchoninic acid (BCA) assay (Thermo Fisher Scientific). Absorbance was measured at a wavelength of 562 nm using a microplate reader (Thermo Fisher Scientific). The proteins (20 μg/well) were separated by electrophoresis with a Mini-PROTEAN^®^ Tetra gel casting (Bio-Rad Laboratories) and then electroblotted onto nitrocellulose membranes using the Trans-blot® Turbo™ Transfer system (Bio-Rad Laboratories). The membranes were stained with Ponceau stain (Sigma-Aldrich Corporation) and blocked with odyssey blocking buffer (LI-COR Biosciences, Lincoln, NE, USA) for 1 h at RT. After washing three times with Tris-buffered saline with 0.1% Tween-20 (TBST) for 15 min, the membranes were incubated overnight at 4 °C while gently shaking with primary antibodies against β-catenin (dilution, 1:5,000; 6B3; Cell Signaling Technology, Inc., Danvers, MA, USA)^92^, β-actin (dilution, 1:2,500; 13E5; Cell Signaling Technology, Inc.)^92^, as an internal control, and LaminB1, as a nucleic protein control (dilution, 1:500; B-10; Santa Cruz Biotechnology, Inc., Dallas, TX, USA)^93^. After washing three times for 15 min, the membranes were incubated for 1 h at RT with secondary antibodies that included goat anti-mouse FITC-conjugated antibody against Lamin B1 (dilution, 1:10,000; ab6785; Abcam, Cambridge, MA, USA) and donkey anti-rabbit Cy3-conjugated antibody against β-catenin and β-actin (dilution, 1:10,000; Jackson ImmunoResearch Laboratories, West Grove, PA, USA). The results were captured using an Amersham™ Imager 680 system (Amersham Biosciences Corporation, Amersham, UK), detected using QuanTL program, and analyzed with ImagaJ software. Lamin B1 and β-actin were used as reference proteins in the nuclear and cytoplasmic fractions, respectively.

### Statistical analysis

All statistical analyses were performed using IBM SPSS Statistics for Mac, version 28.0 (IBM Corporation, Armonk, NY, USA). The Mann–Whitney U test was used for comparisons of two independent groups. A probability (*p*) value of < 0.05 was considered statistically significant (n = 4). All graphics were produced using GraphPad Prism software version 9.0.0 (GraphPad Software, Inc., San Diego, CA, USA).

### Ethical approval

This study protocol was approved by the Human Research Ethics Committee, Faculty of Dentistry, Chulalongkorn University (approval code: HRE-DCU2018/DCU).

## Supplementary Information

Supplementary Figure 1.

Supplementary Figure 2.

Supplementary Figure 3.

Supplementary Legends.

Supplementary Information.

## Data Availability

The RT-qPCR gene expression data has been deposited to the Gene Expression Omnibus (GEO) repository, https://www.ncbi.nlm.nih.gov/geo/query/acc.cgi?acc=GSE225547. The other generated and analyzed datasets during the current study are available from the corresponding author upon reasonable request.
