# Supplementary figures and images for: Dissecting specific Wnt components governing osteogenic differentiation potential by human periodontal ligament stem cells through interleukin-6
Source: Sci Rep. 2023 Jun 3;13:9055. doi: 10.1038/s41598-023-35569-8 (PMC10239497; doi:10.1038/s41598-023-35569-8)

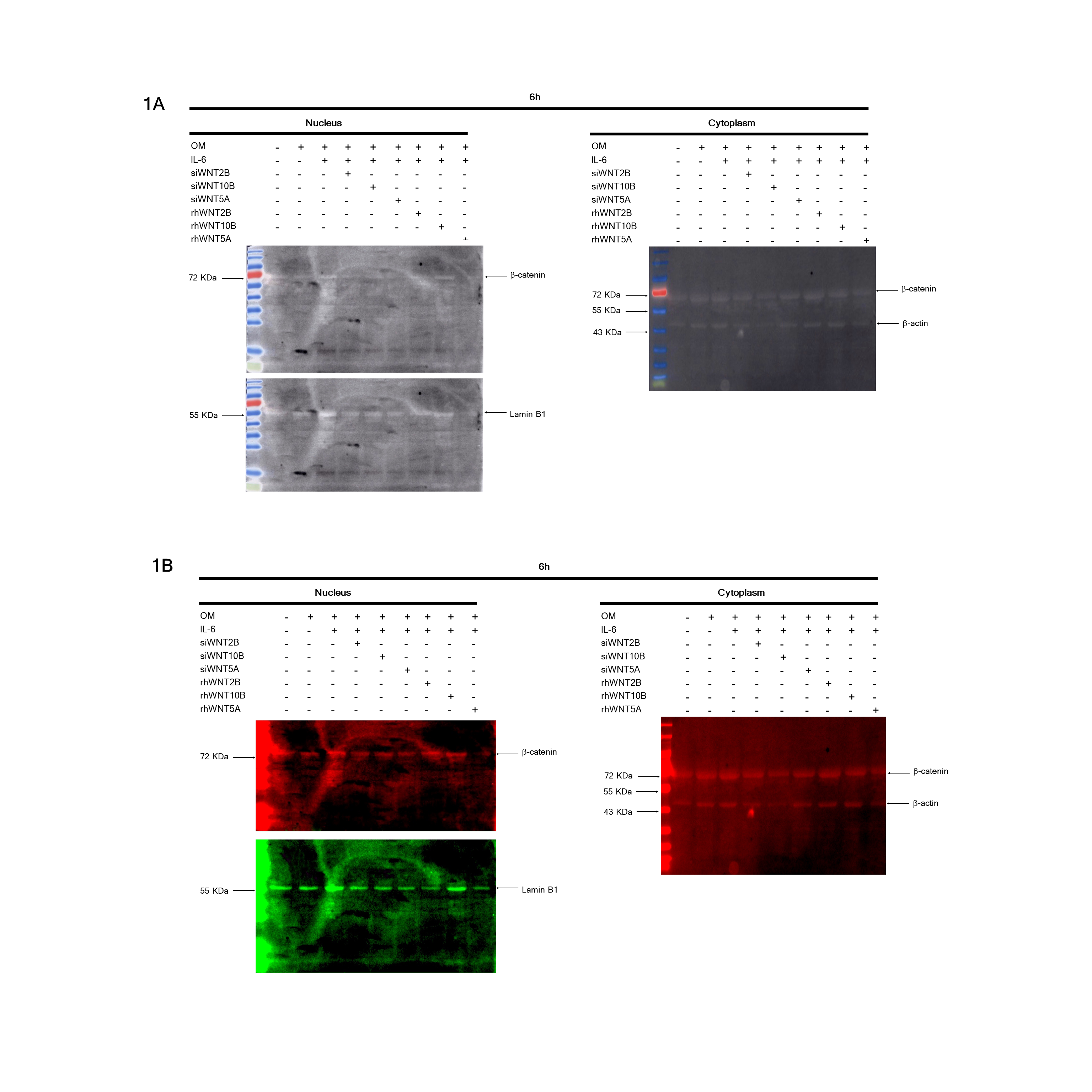

Supplement: Supplementary file 1 — Supplementary Figure 1. [file 41598_2023_35569_MOESM1_ESM.tif]

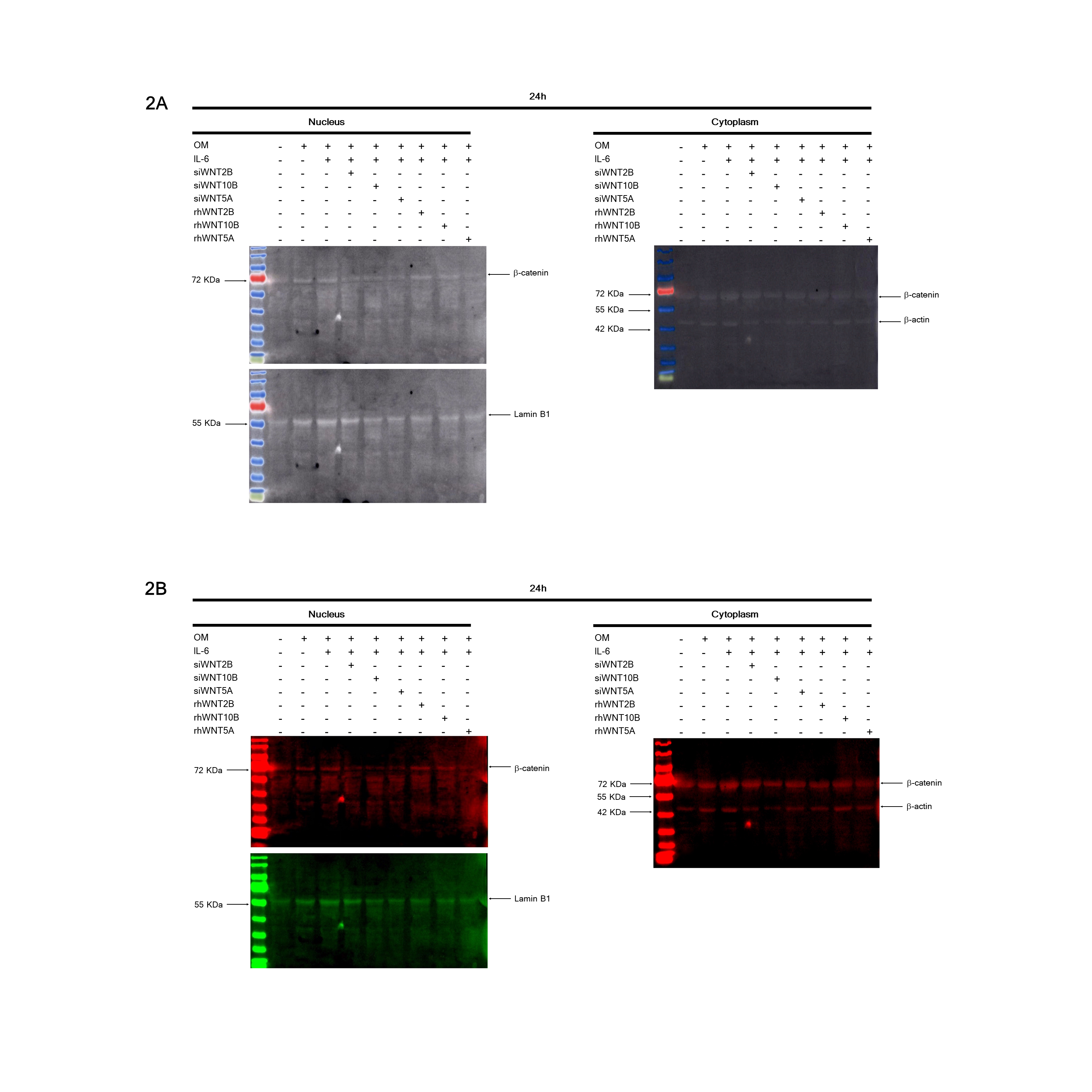

Supplement: Supplementary file 2 — Supplementary Figure 2. [file 41598_2023_35569_MOESM2_ESM.tif]

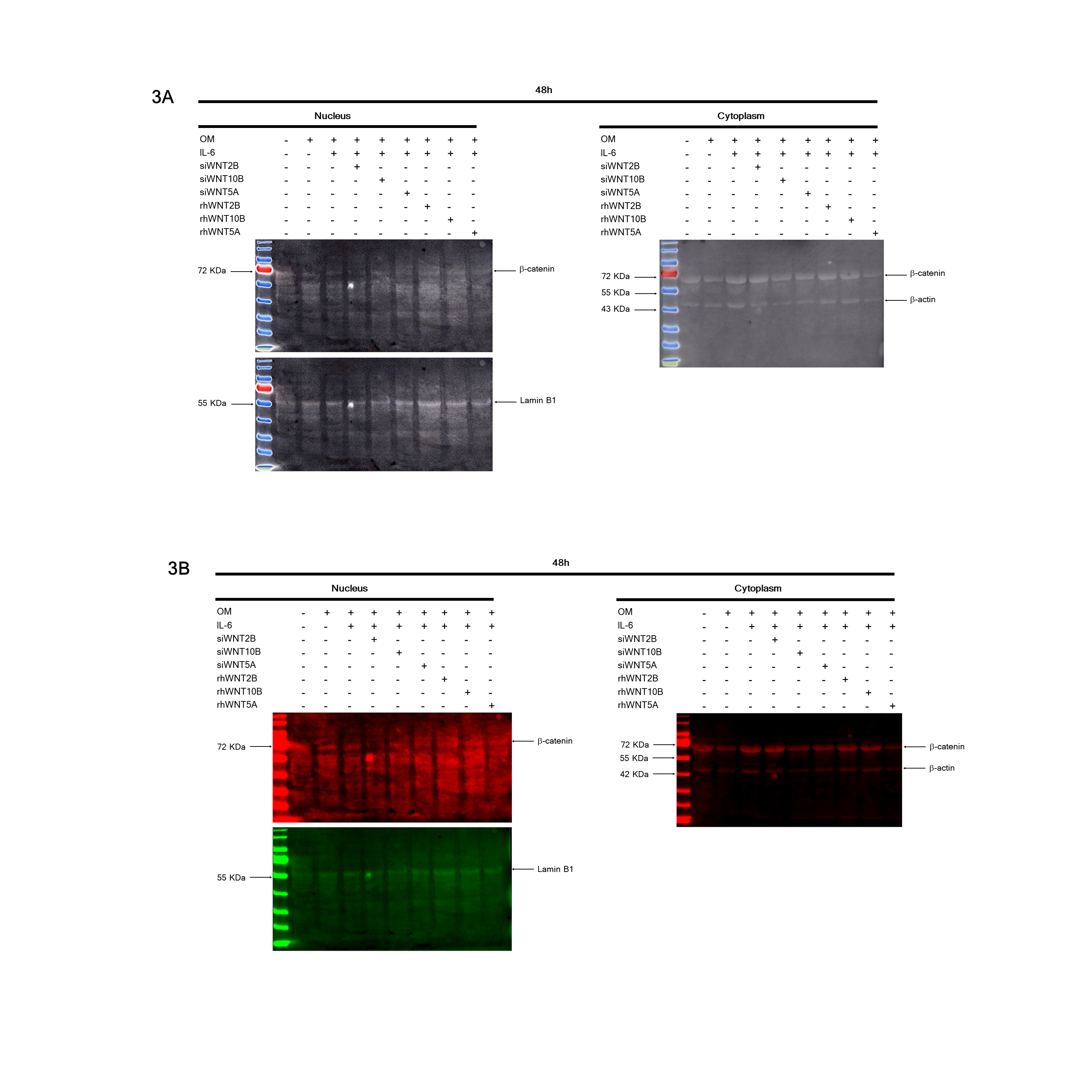

Supplement: Supplementary file 3 — Supplementary Figure 3. [file 41598_2023_35569_MOESM3_ESM.tif]
