# Supplementary material for: Dissecting specific Wnt components governing osteogenic differentiation potential by human periodontal ligament stem cells through interleukin-6
Source: Sci Rep. 2023 Jun 3;13:9055. doi: 10.1038/s41598-023-35569-8 (PMC10239497; doi:10.1038/s41598-023-35569-8)
Supplement: Supplementary file 4 — Supplementary Legends. [file 41598_2023_35569_MOESM4_ESM.docx]

**Supplementary Figure legends**

**Supplementary Figure 1**. Western blotting analysis of β-catenin stabilization and translocation in the nuclear and cytoplasmic fraction under IL-6 treatment at 6 h. (A) The membranes were captured using an Amersham™ Imager 680 system. (B) The signals were detected using QuanTL program.

**Supplementary Figure 2**. Western blotting analysis of β-catenin stabilization and translocation in the nuclear and cytoplasmic fraction under IL-6 treatment at 24 h. (A) The membranes were captured using an Amersham™ Imager 680 system. (B) The signals were detected using QuanTL program.

**Supplementary Figure 3**. Western blotting analysis of β-catenin stabilization and translocation in the nuclear and cytoplasmic fraction under IL-6 treatment at 48 h. (A) The membranes were captured using an Amersham™ Imager 680 system. (B) The signals were detected using QuanTL program.
